# Professeur Jacques Fouad Acar, l’Afrique et l’« auberge espagnole »

**DOI:** 10.48327/BJ0K-9R94

**Published:** 2021-02-18

**Authors:** F.-X. Mbopi-Kéou, L. Bélec

**Affiliations:** 1 Université de Yaoundé I et Faculté de Médicine & des Sciences Biomédicales, Yaoundé, Cameroun; 2 The Institute for the Development of Africa (The-IDA), Yaoundé, Cameroun; 3 UNAIDS Scientific and Technical Advisory Committee (STAC), Genève, Suisse; ^4^ The Board of Health Innovation Exchange, Genève, Suisse; 4 Hôpital européen Georges Pompidou et Université de Paris, Paris Sorbonne Cité, Paris, France

**Keywords:** Diaspora libanaise, Identité, Réseau, Afrique, Résistance aux antibiotiques, Lebanese diaspora, Identity, Network, Africa, Antibiotic resistance

## Abstract

La biographie du professeur Jacques Fouad Acar (1931-2020) montre la trajectoire exceptionnelle d’un médecin atypique, infectiologue-clinicien et microbiologiste, propulsé par la dynamique internationale d’intégration et de dépassement social issue de la diaspora libanaise avec ses premières expériences fondatrices à Dakar au Sénégal, en Afrique Occidentale Française, à l’âge d’or de la médecine coloniale française. L’empreinte de Jacques Acar comprendra trois dimensions remarquables: d’une part, la promotion du raisonnement clinico-biologique multidisciplinaire intégré en pathologie infectieuse; d’autre part, l’indépendance de pensée dans le champ de l’action, qui deviendra son leitmotiv durant sa carrière hospitalo-universitaire, lui permettant d’intégrer « l’esprit de corps pastorien » dans ses recherches fondamentales à l’Institut Pasteur de Paris sur les mécanismes moléculaires de la résistance aux antibiotiques et de participer à l’explosion de la médecine mondiale; enfin, son intelligence affective incomparable potentialisée par son sens instinctif au réseautage, avec des élèves de toutes origines et disciplines.

## Abstract (en)

The biography of Professor Jacques Fouad Acar (1931-2020) shows the exceptional trajectory of an atypical doctor, infectiologist-clinician and microbiologist, propelled by the international dynamics of integration and social progress originating in the Lebanese diaspora with his first founding experiences in Dakar, Senegal, in French West Africa, during the golden age of French colonial medicine. Jacques Acar’s imprint will comprise three remarkable dimensions: on the one hand, the promotion of integrated multidisciplinary clinical-biological reasoning in infectious pathology; on the other hand, independence of thought in the field of action, which will become his leitmotiv during his university hospital career, allowing him to integrate “pastoral esprit de corps” into his fundamental research at the Pasteur Institute in Paris on the molecular mechanisms of antibiotic resistance and to participate in the explosion of world medicine; lastly, his unique emotional intelligence potentiated by his instinctive sense of networking, with students of all origins and disciplines.

## Introduction

« Que peut-on savoir d’un homme aujourd’hui ? » se posait Jean-Paul Sartre à propos de Gustave Flaubert. La même question empreinte de subjectivité et d’historicité pourrait être appliquée au Pr Jacques Fouad Acar, médecin infectiologue-clinicien et microbiologiste émérite aux mille talents (1,2), un homme hors du commun, mort à l’âge de 88 ans en pleine activité, entouré de sa proche famille et du souvenir amical et attentif de ses élèves (2,3,4). Le coup de tonnerre du décès ce grand patron de la microbiologie clinique, le 27 mars 2020, des suites de COVID-19 rapidement évolutif, est aussi la facétie d’un savant mort sur scène lors d’une vie entièrement dédiée à la lutte contre les microbes et dont le dernier paradoxe sera d’être emporté par un virus inédit.

**Fig. 1:**
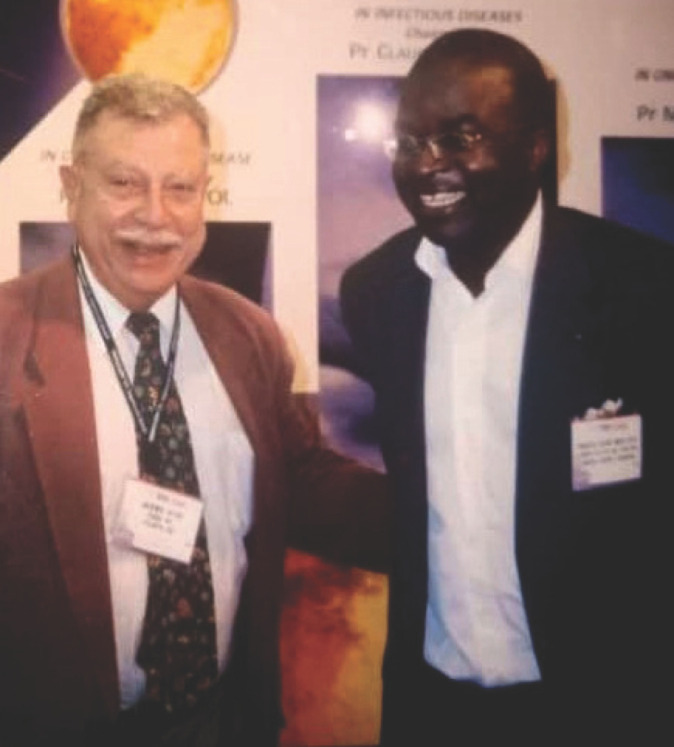
Les professeurs Jacques Acar et François-Xavier Mbopi Kéou à la 39^e^ Interscience Conference on Antimicrobial Agents and Chemotherapy (ICAAC) à San Francisco aux Etats-Unis en septembre 1999 Professors Jacques Acar and François-Xavier Mbopi Kéou at the 39^th^ Interscience Conference on Antimicrobial Agents and Chemotherapy (ICAAC) in San Francisco, USA in September 1999

**Figure:**
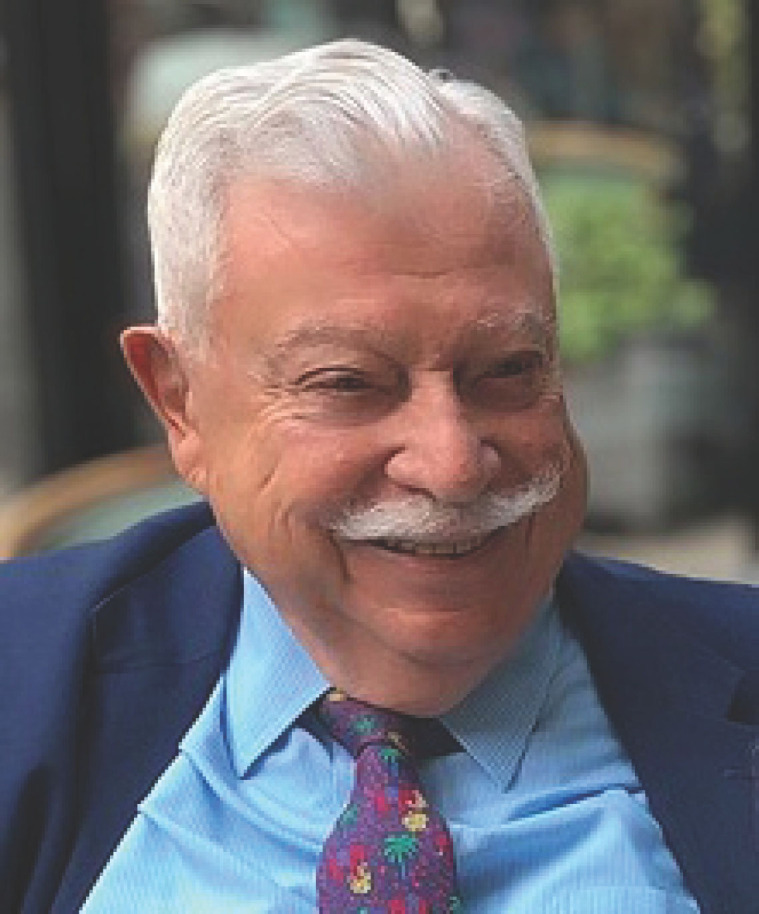


Les premiers éléments mémoriels montrent que Jacques Acar est né le 13 avril 1931 à Dakar, au Sénégal, en Afrique Occidentale Française, de parents libanais originaires de Deir el-Qamar, terre traditionnelle du Mont-Liban d’où les Chrétiens maronites émigraient vers l’Ouest depuis l’Empire ottoman. La naissance de Jacques Acar sur le continent africain et son appartenance à la diaspora libanaise, seront deux éléments majeurs, fondateurs de sa trajectoire personnelle, éducationnelle, relationnelle et professionnelle. L’Afrique coloniale sera la toile de fonds où s’inscriront les premiers repères d’éducation avec sa hiérarchisation culturelle des valeurs. Le Sénégal deviendra très tôt le théâtre des premières expériences fondatrices au sujet des maladies infectieuses, avec des souvenirs terrifiants des épidémies passées de peste dans toute la partie côtière du Sénégal, des périodes angoissantes de fièvre jaune qui déclenchaient des quarantaines de plusieurs mois, sans bateau ni contact avec le reste du monde, ou encore de la vaste campagne de vaccination antiamarile entreprise en 1940. Les maladies infectieuses étaient alors considérées dans l’entourage familial du jeune Acar comme faisant partie de la nature, bonne par essence, mais parfois teintée d’effroi (1). Après son doctorat à l’Ecole de Médecine de l’université de Paris (1949-1954), le Dr Jacques Acar est rattrapé par le Service National, obligatoire, de trente mois, en tant que médecin « appelé » (1955-1958). Il rejoindra pour un court séjour l’Ecole d’application du Pharo à Marseille, où les jeunes médecins appelés recevaient leur formation militaire et leur spécialisation médico-tropicale avant d’aller exercer outre-mer. Par la suite, Jacques Acar sera affecté en Afrique de l’Ouest, notamment à Gao (Mali), comme médecin « de terrain ». Signe prémonitoire, sa route de médecin militaire croisa le chemin du jeune aspirant Marc Gentilini, de peu son aîné, qui deviendra également un pape des maladies infectieuses. Cette expérience d’exercice médical en Afrique sera déterminante pour lui, confortant son approche clinicienne de la pathologie et son attrait pour les maladies infectieuses. Durant cette période, Jacques Acar rencontra à Dakar le futur Pr Didier Raoult (celui qui deviendra un autre pape des maladies infectieuses et de la microbiologie clinique), encore enfant, et son père médecin du Corps de santé colonial qui créa en 1953 l’Office de recherche pour l’alimentation et la nutrition africaine (Orana) à Dakar, à deux pas de l’Institut Pasteur de Dakar et du Centre hospitalier Aristide Le Dantec, et non loin de l’hôpital Principal de Dakar et des plages de l’Anse Bernard. Jacques Acra rencontra également un futur compagnon de route, le Dr Maxime Armengaud, médecin militaire issu de l’École du service de santé des armées de Bordeaux (Santé Navale), affecté à Dakar (hôpitaux de Fann et Le Dantec) en 1955, et qui sera nommé professeur de maladies infectieuses et tropicales en 1958. Au retour de l’armée, Jacques Acar effectuera son internat en Médecine interne, Pédiatrie et Maladies Infectieuses de 1958 à 1962, réalisé en majeure partie à l’hôpital Claude Bernard, Paris, puis son clinicat en Maladies Infectieuses à l’hôpital Claude Bernard de 1962 à 1966.

La famille de Jacques Acar appartient à la diaspora libanaise, dont la présence au Sénégal s’inscrivit dans le mouvement plus large depuis la fin du XIX^e^ siècle, des migrations libanaises, notamment chrétiennes, vers l’Afrique de l’Ouest, lorsque le Sénégal accueillait les premiers immigrants libanais du continent, en escale à Dakar pour embarquer vers les Amériques. La diaspora libanaise de par son ampleur, largement mondialisée, et sa structuration culturelle et économique deviendra un élément déterminant de l’ascension sociale de Jacques Acar. Ses origines libanaises s’inscrivent en effet rapidement dans une perspective précoce de dépassement social offert par le cercle codifié et solidaire de la communauté libanaise de Dakar comme espace de formation et de socialisation. En particulier, la capacité extraordinaire d’adaptation et de reconversion de cette communauté en grande partie constituée d’élites économiques d’origine urbaine permet d’occuper de nouveaux territoires très différents avec suffisamment de souplesse dans leurs relations avec les populations autochtones, pour permettre une insertion rapide et des résultats d’activités intermédiaires comme entrepreneuriales dans des conditions de départ souvent difficiles. Ce sens inédit des valeurs marchandes s’épanouira par la suite, à n’en pas douter, dans les relations complexes que Jacques Acar entretiendra avec les laboratoires pharmaceutiques, notamment dans le domaine du développement des molécules antibiotiques. Cette pratique de l’organisation communautaire en réseaux de la diaspora libanaise semble avoir prédisposé Jacques Acar à l’exercice de formes de communication multiples intra- et trans- nationales, à la recherche d’espaces nouveaux, d’insertion originale, voire d’exterritorialité systématique, que préfigurera son amour des voyages intercontinentaux, sa fascination pour l’Amérique du Nord, et in fine son entrée de plein fouet dans ce qui sera bien plus tard les prémisses de la mondialisation. Les élèves de Jacques Acar s’amusaient à dire à l’envie que leur Maître avait dans ses appartements, en toutes circonstances, deux valises de prêtes, l’une pour les pays chauds et l’autre pour les pays froids. La mobilisation des ressources communautaires et l’utilisation de réseaux de solidarités de premiers cercles, familiaux, amicaux comme professionnels, tiendront par la suite une place prépondérante dans la mobilité sociale de Jacques Acar, avec sa faculté sans précédent de savoir communiquer avec tout le monde et de créer des liens synergiques entre des individus de toutes origines et de toutes formations, laissant chacun s’exprimer selon ses capacités. Dans le domaine professionnel, les réussites du réseautage mis en place par Jacques Acar se traduiront par la création de plusieurs sociétés savantes et scientifiques de renom, et de groupes de travail et réseaux internationaux dans le domaine de la microbiologie clinique. Enfin, le sentiment d’appartenance à une minorité culturelle, devant l’altérité des populations africaines autochtones, ou l’idéologie dominante des colons originaires de métropole, donna à Jacques Acar un cinquième sens inégalable d’ouverture à l’autre, de partage et de tolérance, faisant de la solidarité, à elle seule, un facteur de réussite.

À partir des deux fondements africains et libanais de son existence, la trajectoire personnelle et scientifique de Jacques Acar peut être considérée comme extraordinaire et hors des chemins battus. Trois de ses réussites sont particulièrement remarquables.

La première réussite fut de promouvoir le raisonnement clinico-biologique multidisciplinaire intégré en pathologie infectieuse. Jacques Acar a bénéficié d’un double cursus de formation, en médecine interne et pathologie infectieuse, et en sciences microbiologiques. Le génie de Jacques Acar a été d’aborder les maladies infectieuses dans leur complexité clinico-biologique, en associant systématiquement leurs dimensions clinique et microbiologique, ainsi que la recherche fondamentale, appliquée et translationnelle. Dans le champ pratique, cette approche transversale a été à l’origine du développement des équipes mobiles d’infectiologie, fruit d’une microbiologie médicale engagée « au lit du malade », qui a inspiré des générations de microbiologistes cliniques, comme le Pr Laurent Gutmann qui en parachèvera le modèle à l’hôpital Broussais (Paris) puis à l’hôpital européen Georges Pompidou (Paris).

La seconde réussite concerne son indépendance de pensée dans le champ de l’action. En 1962, Jacques Acar accepta la position de chef de clinique en maladies infectieuses à l’hôpital Claude Bernard, Paris, France. Selon le Pr Didier Raoult, « il refusa d’obéir aux tenants du pouvoir de l’establishment hospitalo-universitaire traditionnel de l’hôpital Claude Bernard » (4). Jacques Acar se réfugia par la suite à l’hôpital St-Joseph de Paris, un hôpital plus périphérique et moins exposé aux dissensions hospitalo-universitaires parisiennes, où il occupa la chefferie du service de bactériologie. Jacques Acar aimait à rappeler et décrire, avec un émoi non feint teinté d’émotions, que l’hôpital St-Joseph de Paris lui rappelait le style architectural colonial de l’hôpital Principal de Dakar, les deux hôpitaux, construits à la même époque, respectivement en 1878 et 1880, possédant une ressemblance remarquable avec leurs pavillons entourant la cour centrale intérieure, tous reliés par des coursives naturellement ventilées et ombragées, bordées à l’envi de gardes corps qui les prolongeaient comme autant de chemins ultimes vers les services de soins et l’aide aux malades. A cette époque, une petite porte exiguë, en apparence incongrue, séparait l’hôpital St-Joseph de l’hôpital Broussais (Paris), et Jacques Acar en devient également chef de service de microbiologie. Dès lors, de son territoire des hôpitaux St-Joseph et Broussais, Jacques Acar mènera son projet loin des sentiers battus hospitalo-universitaires. Parallèlement, Jacques Acar découvrait l’univers de la recherche biomédicale à l’Institut Pasteur de Paris, et y créa son réseau de collaborations scientifiques dans le domaine de la résistance aux antibiotiques, notamment avec le Pr Yves Chabbert (1963-1973). Il intégra cet « esprit de corps pastorien » comme le dénommait Louis Pasteur: ce sentiment d’appartenir à une élite garante de la culture du père-fondateur, ensemble de savoirs et de savoir-faire transmis par compagnonnage, combinant extrême compétence dans la démarche scientifique, l’expérimentation et les pratiques techniques, au bénéfice de l’homme et de son environnement. Dès lors, Jacques Acar s’ouvrait à la médecine mondiale. Dans les années 1990, l’émergence du sida et des hépatites virales nécessita l’implantation d’une unité de virologie médicale et moléculaire à l’hôpital Broussais. Il en confia la mission à un jeune neurologue formé à la Salpêtrière, Paris, le Dr Laurent Bélec, qui venait de passer deux années à l’Institut Pasteur de Bangui en République Centrafricaine durant son service militaire. Alors que le Dr Laurent Bélec était clinicien, Jacques Acar lui fit confiance pour mener sa recherche en tant qu’assistant hospitalo-universitaire à l’Institut Pasteur, Paris, au sein de l’unité d’immunologie microbienne dirigée par le Pr Jacques Pillot, et pour développer la virologie clinique de l’infection à VIH, des hépatites virales et des infections virales opportunistes. Ainsi, Jacques Acar appliquait ses principes de transversalité pour la mixité clinico-biologique à la virologie médicale en légitimant un jeune assistant clinicien sur ses qualités de praticien et sans a priori de sélection négatifs. Jacques Acar se plaisait à dire à ses élèves: « En recherche, j’ai eu beaucoup d’idées; on a copié beaucoup de mes propres idées; les idées n’appartiennent à personne », ou encore « Je ne regarde jamais ailleurs ce que font les autres; par contre, on regarde toujours ce que je fais ». Ainsi Jacques Acar n’avait jamais d’a priori et sa pensée était largement autonome, mais sans prosélytisme. De fait, Jacques Acar devint progressivement une « star internationale » (4), largement reconnue hors des frontières dans la communauté médicale et scientifique mondiale, mais quelque peu marginalisé à Paris (4). En 2001, Jacques Acar en résumait ainsi les enjeux des nouveaux périls infectieux auquel le monde moderne était confronté: « Deux grands événements ont marqué les maladies infectieuses au cours des trente dernières années. D’une part, la renaissance de la discipline par la prise de conscience de l’évolutivité - au sens darwinien - de ces maladies et du risque d’émergence de nouvelles maladies infectieuses (l’épidémie de sida en est un exemple). D’autre part, la contingence de nos attitudes thérapeutiques face au dynamisme du monde microbien, avec des conséquences dramatiques comme l’extension incontrôlée de la résistance aux antibiotiques. »

La troisième réussite concerne son intelligence affective incomparable. Jacques Acar était le chantre du réseautage, de la fédération de proximité comme internationale, de la chaleur humaine, de l’acceptation de toutes les origines et de toutes les disciplines, sans a priori, avec la seule volonté de façonner des alcôves où chacun puisse trouver les conditions de possibilités pour s’y épanouir et exprimer ses capacités. Cette ouverture affective s’affichait d’emblée comme la réminiscence de ses origines libanaises, transcendées par sa voix si unique qui faisait trembler les vitres de son bureau mansardé de l’hôpital St-Joseph, mais sans ébranler sa fidèle secrétaire, aussi dévouée qu’effacée (« Monique, mes fiches !», entendait-on tonner au loin des chiens-assis). Jacques Acar aimait communiquer et parler à tout le monde. Après avoir rencontré ce grand patron, tous ses élèves et collaborateurs étaient touchés en profondeur et en ressortaient transformés comme désormais convaincus de pouvoir donner un sens à leur vie.

Le laboratoire de Jacques Acar accueillait en permanence de nombreux étudiants, chercheurs et stagiaires du monde entier, en provenance du Liban, d’Afrique et d’ailleurs, telle une « auberge espagnole », se plaisait-il à reconnaître. Bien évidemment, l’histoire n’est jamais terminée et continue. Pour paraphraser Flaubert, le tort serait de vouloir conclure.

## Remerciements

Les auteurs remercient le Dr Jean-Marie Milleliri pour son expertise de la médecine coloniale d’avant les indépendances.

## Conflits D'intérêts

Les auteurs ne déclarent aucun conflit d’intérêts.
